# A Favourable Outcome in a Congenital Leukaemia Patient With Unique Cytogenetic Abnormalities

**DOI:** 10.7759/cureus.70345

**Published:** 2024-09-27

**Authors:** Ravindran Ankathil, Nazihah Mohd Yunus, Wan Nur Amalina Zakaria, Salfarina Iberahim, Mohd Ridzuan Hamid, Aziati Azwari Annuar

**Affiliations:** 1 Department of Cytogenetics and Genomics, Jubilee Centre for Medical Research, Jubilee Mission Medical College and Research Institute, Thrissur, IND; 2 Central Research Laboratory, PMS College of Dental Sciences and Research, Trivandrum, IND; 3 Human Genome Centre, School of Medical Sciences, Universiti Sains Malaysia Health Campus, Kubang Kerian, MYS; 4 Genetic Pathology, School of Medical Sciences, Universiti Sains Malaysia Health Campus, Kubang Kerian, MYS; 5 Hematology, School of Medical Sciences, Universiti Sains Malaysia Health Campus, Kubang Kerian, MYS

**Keywords:** b acute lymphoblastic leukaemia, congenital leukaemia, del(7)(q33q35), t(5:15), trisomy 22

## Abstract

Congenital leukaemia (CL) is an exceptionally uncommon hematologic malignancy originating intrauterine and is typically associated with an unfavourable prognosis. The present case is a seven-day-old Malay baby girl who presented with mild fever and hepatosplenomegaly. She was initially treated as neonatal sepsis however subsequent investigations with bone marrow, trephine biopsy and immunophenotyping were consistent with B acute lymphoblastic leukaemia. The peripheral blood smear showed the presence of 90% blast cells. Cytogenetically, she harboured an unusual complex karyotype, 46,XX,der(5) t(5;15)(p15;q15),del(7)(q33q35)/47,idem,+2.ish t(5;15)(wcp15+)+22(wcp22+).

This rare case with extremely atypical cytogenetic findings is being brought to light since the patient responded favourably to the standard chemotherapy Interfant 06 protocol, during which she obtained many episodes of remission, and she still survives after three years of treatment. Despite that, she carries del(7), which is normally associated with adverse outcomes in myeloid disorders, but not in lymphoid disorders; the existence of t(5;15)(p15;q15) could be the element that contributes to her fortunate outcome. Although trisomy 22 is identified as a clonal abnormality, its significance in her case and lymphoid disorders remains unknown and requires further investigation.

## Introduction

Congenital leukaemia (CL), which contributes less than 1% of all childhood leukaemias, is diagnosed either at birth or at any time within 28 days post-delivery of the infant. The prevalence of CL is approximately one in every five million births [1,2]. Etiological factors in CL include chromosomal abnormality, viral infections and maternal exposure to radiation during pregnancy [1,3]. Genetically, a chromosomal abnormality such as trisomy 21 increases the risk of developing acute lymphoblastic leukaemia (ALL) to 20-fold greater than the usual chromosome complement [4]. Fusion of the KMT2A gene is observed in approximately 50% of cases of acute leukaemias in infants [5]. History of fetal loss and high birth weight (more than 4000g) have also been reported as risk factors for infant ALL [6].

## Case presentation

This girl presented on day seven of life with a two-day history of fever. She otherwise had no history of cough, vomiting, diarrhoea, or fits. The basic test showed leukocytosis and thrombocytopenia. At the presentation, she was active and feeding usually. She was born full-term via spontaneous vaginal delivery with a good APGAR score. Antenatal history was uneventful, with no maternal history of exposure to radiation, chemicals, infection, or drug intake during pregnancy. There was no history of cancer or genetic disorders in the family.

At the initial presentation, the girl of non-syndromic appearance was febrile, with a temperature of 37.8°C. She otherwise appeared comfortable. No bruises or other skin lesions, such as leukemic cutis, were noted. There was hepatosplenomegaly, both palpable about 4cm below the subcostal margin. There was no lymphadenopathy. Examination of the cardiovascular, lung and central nervous systems was unremarkable.

The total white count was 97.8 x 10⁹/L, haemoglobin was 14.8 g/dL%, and platelet count was 43 x 10⁹/L. Peripheral blood smear revealed 90% blasts with various sizes seen and consistent with acute leukaemia. Bone marrow aspiration (BMA) showed predominantly blast cells seen on the hemodiluted marrow. Both trephine and bone marrow aspiration were consistent with congenital acute leukaemia (B-cell acute lymphoblastic leukaemia (B-ALL)). Immunophenotyping showed the presence of 66-70% cells at the blast window, which was positive for CD79a, CD19, CD20, CD10, CD38, nTdT and CD58 and cyCD22, the presence of immature B lymphoid cells, which showed maturation arrest suggestive of B-ALL. Toxoplasmosis, rubella cytomegalovirus, herpes simplex, and HIV (TORCH) screening, septic, and molecular tests for the BCR/ABL fusion gene were negatives. The renal and liver function tests were also normal.

**Figure 1:**
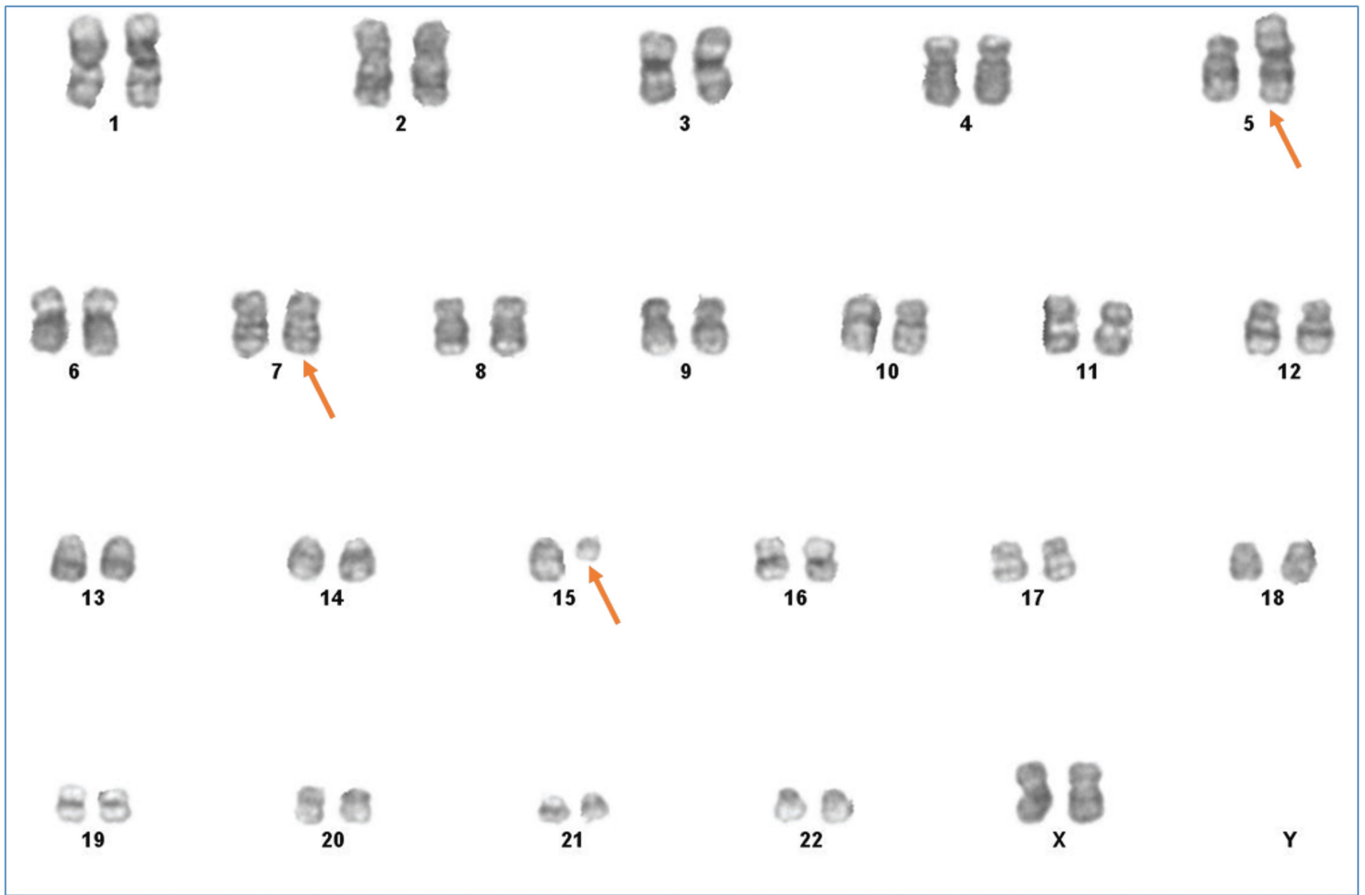
Patient’s bone marrow karyogram shows first abnormal clone with 46,XX,der(5)t(5;15)(p15;q15),del(7)(q33q35)

**Figure 2:**
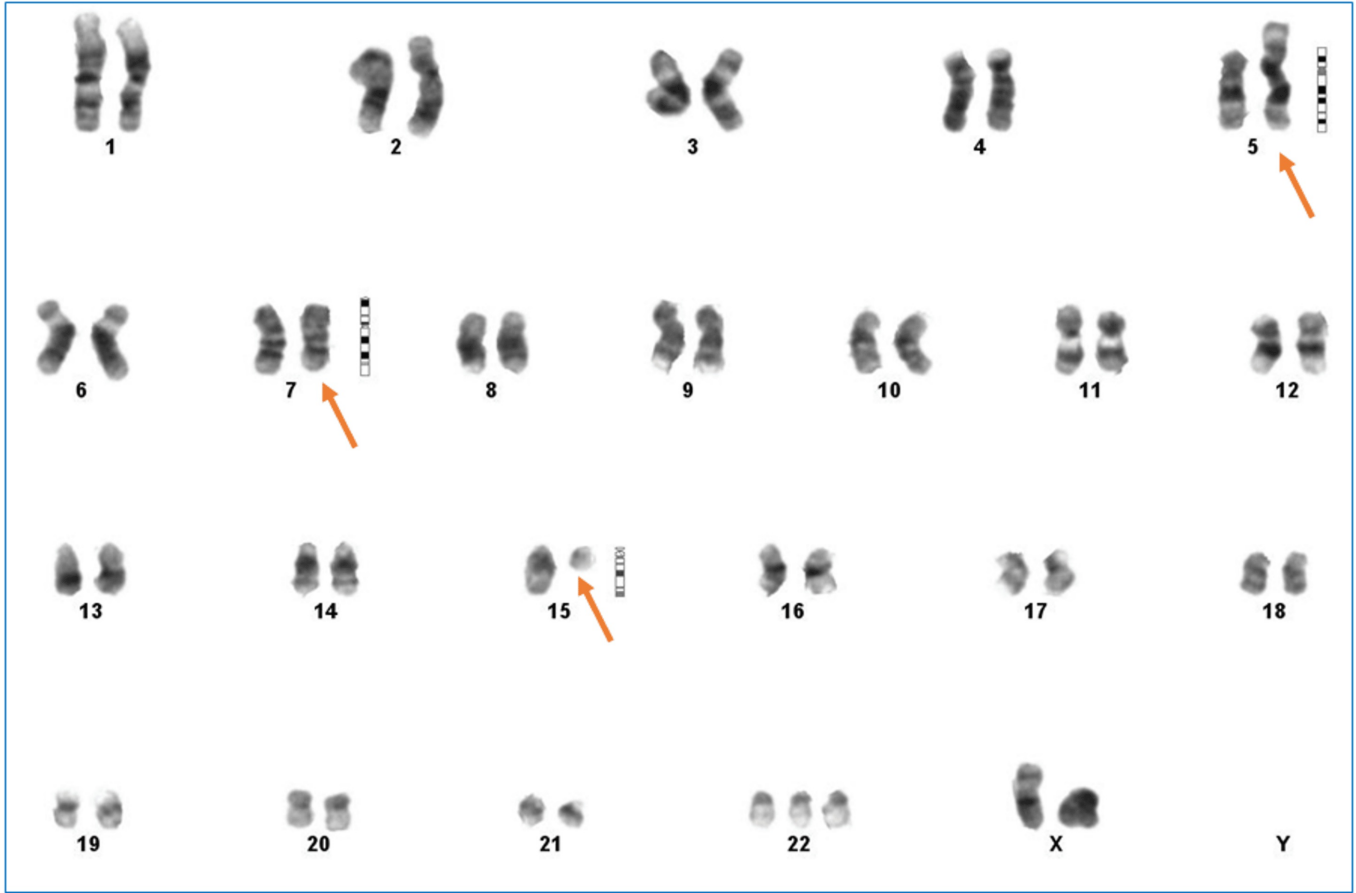
Patient’s bone marrow karyogram showing second abnormal clones with 47, XX der(5)t(5;15)(p15;q15),del(7)(q33q35),+22

**Figure 3:**
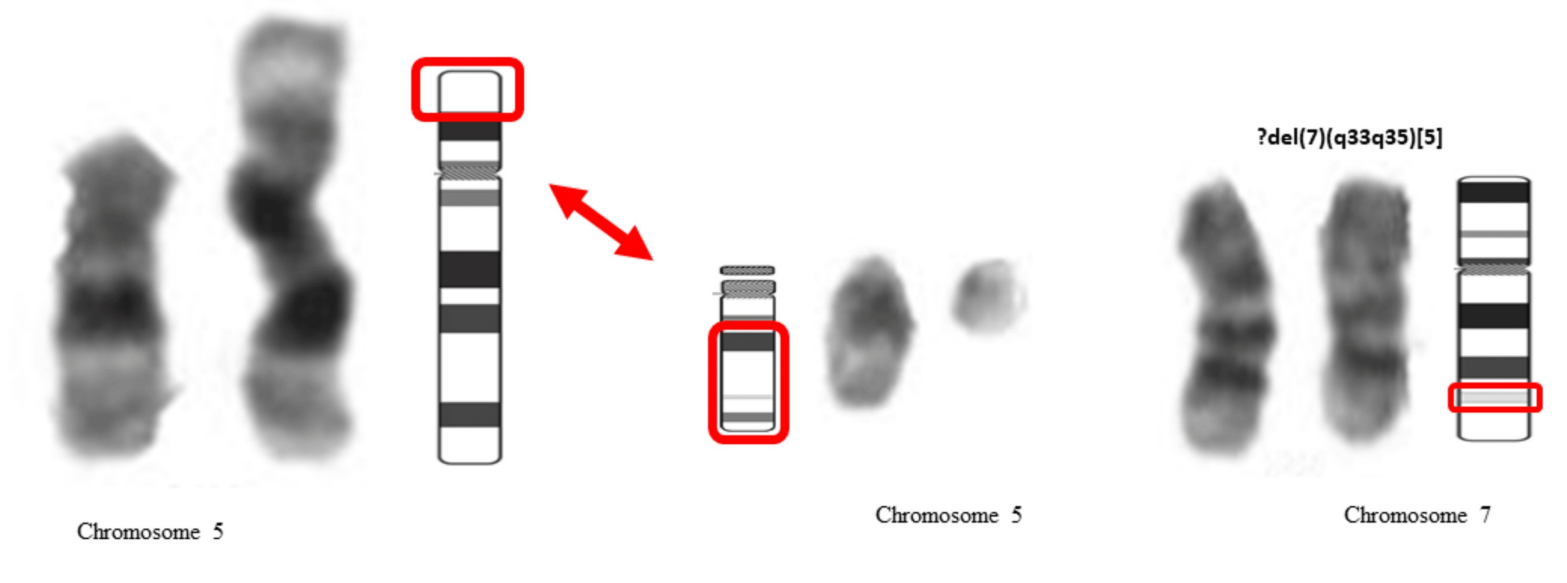
Partial karyotypes from bone marrow metaphases showing translocation between chromosome 5 and 15 and del(7)(q33q35)

**Figure 4:**
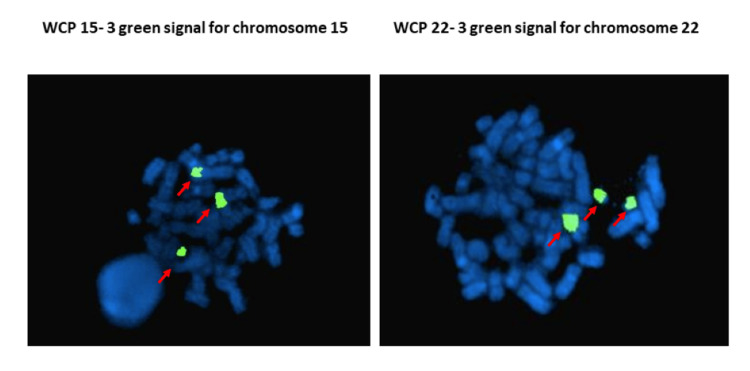
Metaphase fluorescence in situ hybridization (FISH) images of whole chromosome painting probes (WCP) for chromosome 15 showed the presence of three signals for chromosome 15 and three signals for chromosome 22 to confirm the t(5;15) and trisomy of chromosome 22 (trisomy 22).

Conventional cytogenetic analysis and molecular cytogenetic analysis using fluorescence in situ hybridization (FISH) technique were carried out using standard protocols (Figures 1-4).

Bone marrow chromosome analysis revealed two abnormal clones with 46,XX,der(5) t(5;15)(p15;q15),del(7)(q33q35)/47,idem,+22 .ish t(5;15)(wcp15+)+22(wcp22+) karyotype pattern.

The patient was treated with the Interfant Protocol 06 (International Collaborative Treatment Protocol for Infants Under One Year with Acute Lymphoblastic Leukemia) immediately after the diagnosis. Induction was completed after seven weeks and continued with the consolidation phase using protocol 1B for almost one month followed by MARNA (Multi-Agent Regimen for Neonatal Acute Leukemia) and OCTANA D (Optimized Chemotherapy for Treatment of Neonatal Acute Leukemia) protocols. The maintenance phase was embarked on about six months after that. The maintenance chemotherapy was withheld after nine months due to severe thrombocytopenia but was restarted after three months. She completed her maintenance therapy eleven months after that. She is now aged three years, and clinically well. There was no evidence of minimal residual disease (MRD) based on immunophenotyping and the latest BMA examination showed marrow in remission.

## Discussion

The majority of CL are myeloid in origin (56-64%), followed by lymphoid (21-38%) and mixed pattern (biphenotype) (3-4%), in contrast to childhood leukaemias which are usually lymphoid [3]. Molecularly, the most frequently occurring chromosomal aberration in both neonatal acute myeloid leukaemia (AML) and ALL is a rearrangement of the KMT2A gene, located at chromosome 11q23. There are almost 120 known fusion partners of KMT2A reported in infant leukaemia previously. The most frequently observed cytogenetic/molecular abnormality in non-Down syndrome neonatal leukaemia is t(4;11)(q21.3;q23.3)/KMT2A-AFF1 followed by t(1;22)(p13.3;q13.1)/RBM15-MKL1 and t(8;16)(p11.2;p13.3)/KAT6A-CREBBP [2]. The t(4;11)(q21.3;q23.3)/KMT2A-AFF1 fusion is responsible for approximately 50% of cases of infant acute leukaemias [5]. The other known variant 11q23.3/KMT2a translocation that has been reported in congenital B lineage ALL include t(9;11)(p21.3;q23.3)/KMT2a-MllT3 and t(11;19)(q23;p13). Cytogenetically, the 11q23 or KMKT2A rearrangement was not seen in this case.

CL carries a poor prognosis and is almost always lethal without chemotherapy with only 23% of the overall survival rate before 2000 [7,8]. Generally, factors that trigger poor outcomes include marked leukocytosis, central nervous system (CNS) involvement, massive hepatosplenomegaly, thrombocytopenia, hypogammaglobulinemia and presence of disseminated intravascular coagulation (DIVC).

The clinical presentations for congenital leukaemia are varied. At birth, they may present with fever, hepatosplenomegaly, or symptoms due to low haemoglobin/platelets such as pallor and lethargy, petechiae, or ecchymosis. Some neonates may develop leukemic cutis or chloroma if the leukemic cell infiltrates the skin and this presentation is common in AML, especially in premature babies [9]. Infiltration to the lung might cause respiratory distress or atelectasis [1].

This patient presented with low-grade fever, hyperleukocytosis, and thrombocytopenia. Her congenital leukaemia was evidenced by the proliferation of immature white cells in the peripheral and also in the bone marrow. Our patient’s bone marrow and peripheral blood examination showed the presence of blasts with severe thrombocytopenia. Bone marrow and flow cytometry examination of the blast were confirmed as B-ALL. Investigations excluded the presence of any other disease that can cause a leukemoid reaction that mimics leukemia such as TORCH infection, congenital syphilis, and blood group incompatibility. In cases of Down's syndrome, which can be excluded in this case, the non-malignant proliferation of hematopoietic cells termed transient abnormal myelopoiesis (TAM), might also mimic congenital leukaemia. TAM generally reverts voluntarily and requires only conservative management and a phase of monitoring [10].

Previous studies had documented a few different types of cytogenetic abnormalities in congenital leukaemia. These include recurrent cytogenetic abnormalities such as t(8;16)(p11.2; p13.3)/KAT6A-CREBBP and t(8;21)(p11;q22), and non-recurrent cytogenetic abnormalities such as t(5;9), deletion of 14p, and deletion of 8p in small number of patients . Few studies identified other abnormalities such as monosomy 7, trisomy 9, trisomy 21, t(9;18), t(11;19), t(9;ll) and t(X;6) [1,7].

Contrary to the above, our patient harboured unique cytogenetic findings. A few double minutes were seen in her peripheral blood taken at diagnosis. Homogenously staining regions (HSRs) or double minute chromosomes (dmin) are small fragments of extrachromosomal DNA seen in some human tumours including lung, ovary, breast, colon, and neuroblastoma [11]. Dmin and HSRs play an important role in tumour cell genetics since they are frequently found to be associated with the overexpression of oncogene products, comprising one of the cytogenetically visible signs of gene amplification.

Apart from that, this patient was noted to have translocation t(5;15)(p15;q11). This unique chromosomal rearrangement has never been reported in congenital leukaemia before. Nevertheless, it has been reported in infant leukaemia where it was categorized as rare and sporadic. So far, there have been about seven cases of infant ALL harbouring t(5;15)(p15;q11-13) [12-15]. This special subgroup of infant ALL achieved a 100% complete remission rate and event-free survival of about 70%. They are characterized by pre-B L1 ALL, CD10 positive, and with a relatively good prognosis as compared to the presence of KMT2A rearrangement cases [12].

Apart from translocation t(5:15), this girl showed deletion 7q23 on one chromosome 7, which is also considered a novel finding in congenital leukaemia. Monosomy of chromosome 7(−7) and del(7q) are recurrent cytogenetic abnormalities that are frequently associated with myelodysplastic syndrome (MDS) and AML and they carry an adverse outcome. The regions found commonly deleted in MDS are 7q22, 7q32-33, and 7q35-36, almost similar to the region in this case. A few genes on chromosome 7 that may play a crucial role in the disease pathogenesis and phenotype of cancers are EZH2, KMT2A, SAMD9, SAMD9L, and CUX1 [16]. 

This girl also harboured trisomy 22 in a majority of her bone marrow metaphases and was confirmed by FISH by using whole chromosome painting probes (WCP) for chromosome 22. It is considered a novel finding in congenital leukaemia cases. Trisomy 22 is an uncommon chromosomal abnormality in acute myeloid leukaemia. Previous studies have shown a correlation between trisomy 22 and acute myeloid leukaemia with a monocytic component, namely acute myelomonocytic leukaemia with marrow eosinophilia [17].

## Conclusions

Congenital leukaemia is an extremely uncommon disease. Despite being mostly associated with poor prognosis, our case however showed a good response to chemotherapy, a favourable foresight. It is reasonable to suggest that the rare cytogenetic aberrations especially t(5;15) might contribute to her favourable prognosis and response to treatment. It is imperative to conduct early cytogenetic analysis, as specific chromosomal rearrangements can have a prognostic impact and may affect the choice of treatment.
